# Line focus x-ray tubes—a new concept to produce high brilliance x-rays

**DOI:** 10.1088/1361-6560/aa910b

**Published:** 2017-10-27

**Authors:** Stefan Bartzsch, Uwe Oelfke

**Affiliations:** 1 The Institute Of Cancer Research, 123 Old Brompton Road, London SW7 3RP, United Kingdom; 2 Department of Radiation Oncology, Klinikum rechts der Isar, Technical University of Munich, Ismaninger Straße 22, 81675 Munich, Germany; stefan.bartzsch@tum.de; uwe.oelfke@icr.ac.uk

**Keywords:** microbeam radiation therapy, x-rays, compact radiation sources, high dose rate, phase contrast imaging, coherence

## Abstract

Currently hard coherent x-ray radiation at high photon fluxes can only be produced with large and expensive radiation sources, such as 3${\rm rd}$ generation synchrotrons. Especially in medicine, this limitation prevents various promising developments in imaging and therapy from being translated into clinical practice. Here we present a new concept of highly brilliant x-ray sources, line focus x-ray tubes (LFXTs), which may serve as a powerful and cheap alternative to synchrotrons and a range of other existing technologies. LFXTs employ an extremely thin focal spot and a rapidly rotating target for the electron beam which causes a change in the physical mechanism of target heating, allowing higher electron beam intensities at the focal spot. Monte Carlo simulations and numeric solutions of the heat equation are used to predict the characteristics of the LFXT. In terms of photon flux and coherence length, the performance of the line focus x-ray tube compares with inverse Compton scattering sources. Dose rates of up to 180 Gy ${\rm s}^{-1}$ can be reached in 50 cm distance from the focal spot. The results demonstrate that the line focus tube can serve as a powerful compact source for phase contrast imaging and microbeam radiation therapy. The production of a prototype seems technically feasible.

## 1. Introduction

Since its discovery by Konrad Röntgen in 1895, x-ray radiation has been established as a versatile tool in science, medicine and industry. The field of x-ray applications in medicine and biology is rapidly evolving and various new developments such as high resolution x-ray imaging (Tafforeau *et al* 2006), phase contrast imaging (Momose *et al* 1996) and microbeam radiation therapy (MRT) (Slatkin *et al* 1992) demand for radiation sources of high brilliance, a quantity that measures the number of photons N emitted per time ${\rm d}t$, from an area ${\rm d}A$, within an emission angle interval ${\rm d}\Omega$ per frequency interval ${\rm d}\nu$.

In conventional medical x-ray imaging contrast arises from relative differences in the absorption coefficient, which are usually low in soft tissue. The relative differences in the refractive index are significantly larger and can be visualized in phase contrast imaging (Pfeiffer *et al* 2006). Various methods have been proposed for phase contrast imaging (Momose 2003), but all are based on the interferometric measurement of phase shifts induced by refractive index variations in the imaged object. The prerequisite to observe interference is the coherence of the radiation source. While temporal coherence can be obtained with the aid of crystal monochromizers, spatial coherence is of particular importance as path length differences between photons emitted from different parts of the x-ray source have to be much smaller than the wavelengths *λ* measuring in the order of only $10^{-11}$ m for hard x-rays. High photon rates at small source sizes are therefore essential in phase contrast imaging.

**Figure 1.**
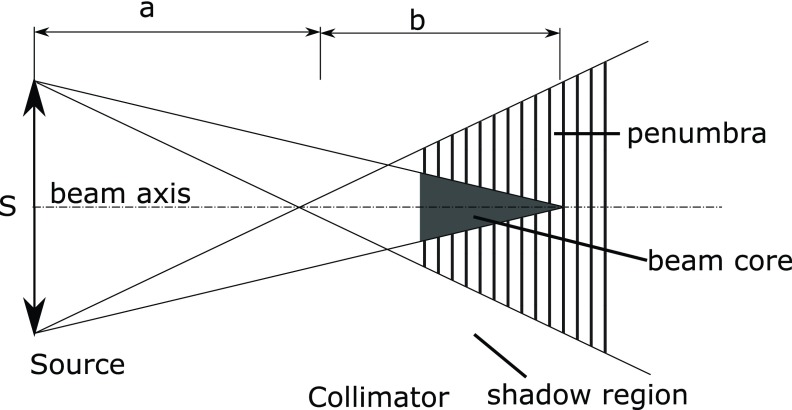
Beam geometry for the collimation of microbeams: in order to produce microbeams with high beam intensities the source size (S) has to be smaller than the collimator aperture. A large source to collimator distance (a) will keep the width increase with distance from the collimator (b) small.

Another medical application demanding for high hard x-ray beam brilliance is microbeam radiation therapy (Slatkin *et al* 1992). This innovative treatment approach in radiation oncology employs arrays of planar micrometre wide x-ray beams with unconventionally high radiation doses of several hundred Grays, separated by a few hundred micrometre wide low dose regions for the treatment of tumours. In numerous preclinical studies MRT was shown to effectively spare irradiated healthy tissue surrounding the tumour target (Laissue *et al* 2001, Serduc *et al* 2008, Bouchet *et al* 2010) on the one hand while efficiently controlling the tumour (Laissue *et al* 1998, Regnard *et al* 2008, Bouchet *et al* 2010) on the other hand. In order to collimate micrometre wide beams, parallel radiation fields and source diameters smaller than the beam width are required as illustrated in figure 1. Furthermore the treatment dose has to be administered at very short time scales to avoid blurring of the MRT dose pattern due to cardiovascular or respiratory motion of the irradiated tissue. Microbeam peak dose rates of more than 100 Gy ${\rm s}^{-1}$ are required. At the moment such dose rates can only be achieved at large 3${\rm rd}$ generation synchrotrons such as the European Synchrotron Radiation Facility (ESRF) in Grenoble, France.

The current ultimate source of brilliant x-rays are 3${\rm rd}$ generation synchrotrons. However, these synchrotrons are large, expensive facilities measuring hundreds of metres across which are often impractical to facilitate a wide-spread application of hard x-rays in science or medicine. In particular MRT and phase contrast imaging have not been established in clinical practice due to the lack of compact high-brilliance x-ray sources (Bech *et al* 2010).

Although, recently developed x-ray sources based on inverse Compton scattering may become an attractive alternative to synchrotrons (Graves *et al* 2009, Variola 2011) the application of this technology is still severely limited by its achievable photon energies, field sizes and dose rates.

Compared to synchrotrons and inverse Compton scattering sources conventional x-ray tubes are a cheap, easily manageable source of x-rays and they are ubiquitously employed, be it in computed tomography, airport security screening or non-destructive material testing. In an x-ray tube electrons are accelerated with a high voltage of up to several 100 kV before they hit an anode target. There only roughly 1$\%$ of their kinetic energy is used to produce divergent, polychromatic x-rays emitted from a usually millimetre sized focal spot, while the remaining 99$\%$ of their energy is converted to heat. Due to beam divergence, broad photon energy spectra, large focal spot widths and a low electron to photon conversion efficiency, the brilliance of radiation produced with x-ray tubes is around 10 orders of magnitude lower than with synchrotrons.

The main limitation in generating highly brilliant x-ray beams based on this conventional technology is the required cooling of the target if a small focal spot is hit by a high intensity electron beam. Various technical developments have aimed to overcome this limitation. Rotating anode x-ray tubes (Pohl 1914, Silbermann 1954) allow the production of considerably higher photon fluxes than x-ray tubes with stationary anodes, but the focal spot size is too large for applications such as MRT and phase contrast imaging. On the other hand microfocus (Liu *et al* 2006) and metal jet x-ray tubes (Hemberg *et al* 2003, Tuohimaa *et al* 2007) provide much smaller focal spots but their photon flux and, for metal jet x-ray tubes, also the photon energy are limited by the tolerable heat load imposed at the small electron focal spots of the target.

To overcome this problem we propose a new technological paradigm based on conventional x-ray tubes by introducing the concept of line-focus x-ray tubes (LFXTs), capable of increasing the beam brilliance by more than two orders of magnitude when compared to conventional x-ray tubes.

## 2. Methods

### 2.1. A new concept of brilliant x-ray generation

**Figure 2.**
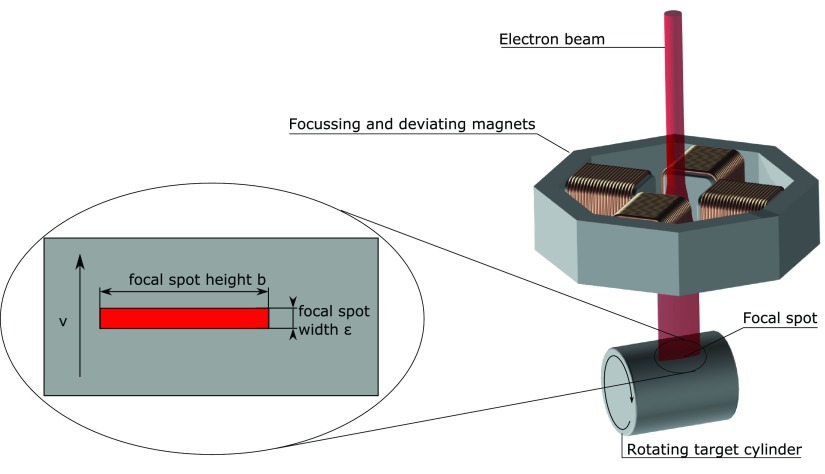
Assembly of the line focus tube (LFXT): electrons are generated, accelerated, focused and hit a rotating target cylinder in a focal spot of high aspect ratio $ \newcommand{\e}{{\rm e}} b/\epsilon$.

The LFXT concept illustrated in figure 2, comprises the generation, acceleration and electromagnetic shaping of an electron beam that hits a fast rotating cylinder target in a thin focus line, i.e. a focal spot, with a very large aspect ratio $ \newcommand{\e}{{\rm e}} b/\epsilon$, where *b* and ϵ refer to the length and width of the focal spot, respectively. While this design looks very similar to the conventional x-ray tube, the LFXTs operates in a so far undescribed physical limit of target heating at high surface velocities and small focal spot widths which we term the heat-capacity limit. This novel physical limit described below permits a significant increase in electron beam current density without raising the focal spot temperature above the melting point of the target.

In conventional rotating anode x-ray tubes heat conduction limits the temperature increase in the focal spot. An electron beam power *P*_cond_ is absorbed at a focal spot surface area $ \newcommand{\e}{{\rm e}} A=b\epsilon$ and almost completely converted into heat. The heat is dissipated by heat conduction and the focal spot temperature increase $\Delta T$ during an exposure time $\Delta t$ is proportional to the electron beam intensity $P_{\rm cond}/A$ at the focal spot (Oosterkamp 1948, Oppelt *et al* 2005),

1 \begin{align} \newcommand{\e}{{\rm e}} \displaystyle \Delta T = \frac{2P_{\rm cond}}{A}\sqrt{\frac{\Delta t}{\pi k\rho c}} \label{eqn:DeltaT} \nonumber \end{align}

where *k*, *ρ* and *c* denote heat conductivity, mass density and heat capacity of the target material. For a rotating target with a surface velocity *v*, $\Delta t$ will be $ \newcommand{\e}{{\rm e}} \epsilon/v$ and therefore, assuming a fixed maximum temperature rise $\Delta T_{\rm max}$ the target can withstand, the maximum electron beam power is

2 \begin{align} \newcommand{\e}{{\rm e}} \displaystyle \begin{array}{@{}rcl@{}} P_{\rm cond}&amp;=&amp;\gamma_1b\sqrt{v\epsilon} \nonumber \\ \gamma_1&amp;=&amp;\frac{1}{2}\Delta T_{\rm max}\sqrt{\pi k \rho c}. \end{array} \label{eqn:PCond} \nonumber \end{align}

Equation (1) as a solution of the heat equation with Neumann boundary conditions assumes that surface heating is the only process of energy transport within the anode. Energy transport by electrons in the target material is completely ignored. However, this is a valid assumption only as long as the heat diffusion length *l*_d_ during electron beam exposure $\Delta t$,

3 \begin{align} \newcommand{\e}{{\rm e}} \displaystyle l_{\rm d}=2\sqrt{\frac{k\Delta t}{\rho c}}=2\sqrt{\frac{k\epsilon}{\rho c v}} \nonumber \end{align}

is much larger than the electron range *l*_e_, $l_{\rm d}\gg l_{\rm e}$. This however, will change for large surface velocities *v*, narrow spot widths ϵ and large electron penetration depths at high acceleration voltages. If we consider the extreme limit where the electron range *l*_e_ is significantly larger than the heat diffusion length *l*_d_, $l_{\rm e}\gg l_{\rm d}$, the heating of the target material is limited by the heat capacity only. A volume element $\delta V$ receiving the heating power $\delta P$ by electron absorption, will heat according to

4 \begin{align} \newcommand{\e}{{\rm e}} \displaystyle \delta P\,\Delta t = \rho\, \delta V c\, \Delta T . \nonumber \end{align}

For a fixed maximum temperature increase $\Delta T_{\rm max}$ this leads, in contrast to equation (2), to a maximum electron beam power of

5 \begin{align} \newcommand{\e}{{\rm e}} \displaystyle \begin{array}{@{}rcl@{}} P_{\rm cap}&amp;=&amp;\gamma_2 v b d \nonumber \\ \gamma_2&amp;=&amp;\rho c \Delta T_{\rm max}. \end{array} \label{eqn:PCap} \nonumber \end{align}

The electron penetration depth *d*, which is defined in the next paragraph, depends on the electron beam energy and the anode material. Importantly, *P*_cap_ does not depend on the focal spot width ϵ anymore. Hence a reduction in focal spot width does not impact on the maximum possible electron beam power. This leads to the remarkable fact that the intensity $P_{\rm cap}/A$ of the electron beam can be increased ad libitum by reducing the focal spot width and is only limited by lateral scattering of electrons in the target which is approximately given by $ \newcommand{\e}{{\rm e}} \epsilon_{\rm min}\approx d/3$ (see also discussion below).

The transition from the conventional heat conduction limit to the heat capacity limit occurs when $P_{\rm cap}=P_{\rm cond}$. The surface velocity *v*_t_ at this transition is

6 \begin{align} \newcommand{\e}{{\rm e}} \displaystyle v_{\rm t}=\frac{\pi k}{4 \rho c}\cdot\frac{\epsilon}{d^2}, \label{eqn:transitionvelocity} \nonumber \end{align}

and the maximum possible increase in brightness, as compared to the heat conduction limit, is equal to the ratio of *P*_cap_ and *P*_cond_ at the smallest possible focal spot width $ \newcommand{\e}{{\rm e}} \epsilon_{\rm min}$,

7 \begin{align} \newcommand{\e}{{\rm e}} \displaystyle \frac{P_{\rm cap}}{P_{\rm cond}}=6\sqrt{\frac{\rho c}{\pi k}}\cdot\sqrt{\epsilon_{\rm min}v}. \nonumber \end{align}

Close to the transition between the two heat transport limits, where electron range and heat diffusion length are similar ($l_{\rm e}\approx l_{\rm d}$), both, heat conduction and electron scattering contribute to the energy transport from the target surface. The maximum electron beam power will be higher than described in either limit (equations (2) and (5)).

### 2.2. Derivation of heat transport in the heat capacity limit

In this paragraph we derive the tube heating in the heat capacity limit, i.e. we assume no heat transport during the time of heating. In practice both, heat conduction and electron energy transport will contribute to the heat dissipation. Especially at conditions where the heat diffusion and the electron range are of similar size the temperature increase at the focal spot will in practice be lower than calculated by either of the two models.

**Figure 3.**
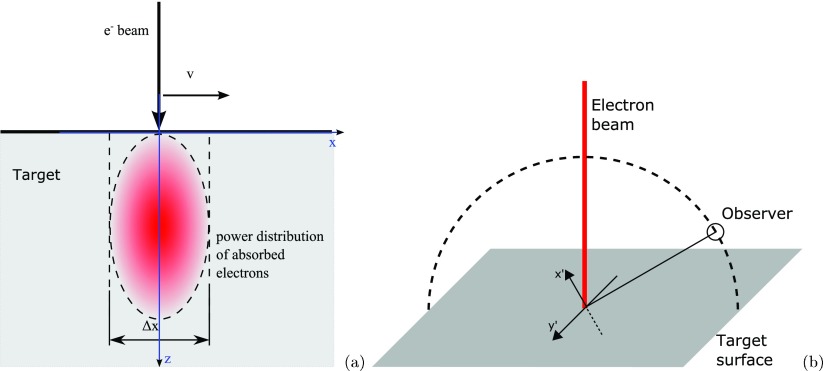
Electron beam energy absorption in the target material. (a) shows the geometry at the anode surface. (b) shows the geometry used for a Monte Carlo study of the focal spot size. An electron beam hits the surface of the tungsten target.

Figure 3(a) shows the geometry in the LFXT. The electron beam moves with velocity *v* along *x* relative to the target surface. Electrons are statistically scattered and absorbed in the target material and create a power distribution $\delta P/\delta V(x, y, z)$. This distribution is time dependent, since the beam is moving along *x*, i.e. $x = x(t)$. In the heat capacity limit, the total temperature increase $\Delta T$ that a volume element $\delta V$ will experience can be calculated via

8 \begin{align} \newcommand{\e}{{\rm e}} \displaystyle \int_{-\Delta t/2}^{\Delta t/2}\frac{\delta P}{\delta V}(x(t), y, z){\rm d}t = \rho c \Delta T \label{eqn:S2} \nonumber \end{align}

We assume that the height *b* of the beam (figure 2) is much larger than the electron scattering range and that $\delta P/\delta V$ does not depend on *y* and

9 \begin{align} \newcommand{\e}{{\rm e}} \displaystyle \begin{array}{@{}rcl@{}} \frac{\delta P}{\delta V}(x(t), y, z) &amp;=&amp; \frac{1}{b}\frac{\delta^2P}{\delta x \delta z}(x(t), z) \nonumber \\ &amp; = &amp; \frac{\dot{N}_{\rm el}}{b}\left&lt;\frac{\delta^2 E_{\rm el}}{\delta x \delta z}(x(t), z)\right&gt;. \end{array} \nonumber \end{align}

The chevrons $\left&lt;\ldots\right&gt;$ are used to denote the ensemble average of all electrons. We have replaced the power *P* by the number of electrons per time $\dot{N}_{\rm el}$ times the average energy absorption *E*_el_ per electron. Integration in equation (8) can be carried out leading to

10 \begin{align} \newcommand{\e}{{\rm e}} \displaystyle \begin{array}{@{}rcl@{}} \frac{\dot{N}_{\rm el}}{vb}\int_{-\Delta x / 2}^{\Delta x /2}\left&lt;\frac{\delta^2E_{\rm el}}{\delta x \delta z}(x, z)\right&gt;{\rm d}x&amp;=&amp;\frac{\dot{N}_{\rm el}}{vb}\left&lt;\frac{\delta E_{\rm el}}{\delta z}(z)\right&gt; \nonumber \\ &amp;=&amp;\frac{P}{vb E_{\rm el}}\left&lt;\frac{\delta E_{\rm el}}{\delta z}\right&gt; \nonumber \\ &amp;=&amp;\rho c \Delta T \end{array} \nonumber \end{align}

The maximal temperature increase is given where $\left&lt;\frac{\delta E_{\rm el}}{\delta z}(z)\right&gt;$ reaches its maximum and hence we identify the quantity $E_{\rm el}/\left&lt;\frac{\delta E_{\rm el}}{\delta z} (z)\right&gt;_{\rm max}$ with the electron penetration depth *d*, which leads to equation (5).

### 2.3. Modifications of the LFXT for MRT

**Figure 4.**
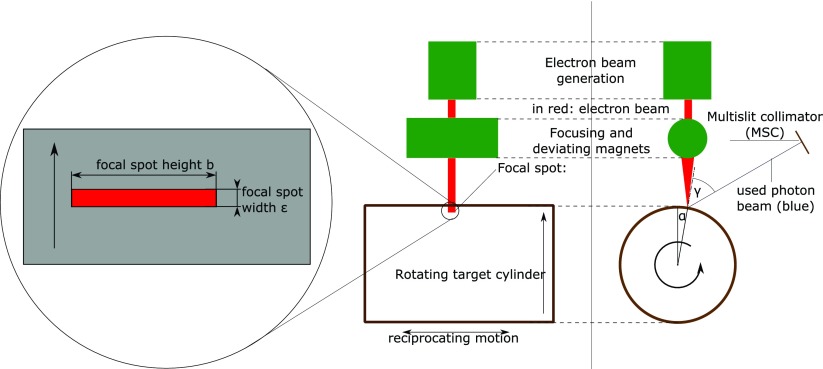
The microbeam tube: the extension of the set-up shown in figure 2 has an an additional multislit collimator (MSC) and a longer tungsten target cylinder that performs a reciprocating motion along the axis in addition to the rotation.

To use the LFXT for the generation of microbeams we modify the set-up in figure 2 to that shown in figure 4, that we further refer to as microbeam tube (MBT), where a multislit collimator (MSC) is added in 0.5 m distance from the focal spot. The MSC has multiple 50 *μ*m wide and 30 mm high slit apertures with a pitch of 400 *μ*m. The slits are not parallel to each other, but account for the beam divergence.

The target cylinder has a radius of 10 cm, a length of 30 cm and rotates at a speed of 17 000 rpm around its axis while performing a reciprocating motion along the axis. Electrons are accelerated up to a kinetic energy of 600 keV and hit the target in a 30 mm high and 100 *μ*m wide focal spot. The incidence angle *α* of the electron beam and the emission angle *γ* are 10 and $60^\circ$. The photon beam is filtered by 1 mm aluminum.

The reciprocating motion will not add a significant increase in the surface velocity and therefore not impact on the maximum achievable dose rate. However, the heat load is distributed over a larger surface area and hence the exposure time can be extended. This is important for MRT treatments, where peak entrance doses of several hundred Grays are applied.

### 2.4. Simulation of the target heating

The optimization of heat dissipation from the target of x-ray tubes led to numerous technical innovations in the past. It is beyond the focus of this work to discuss the various designs, their applicability and performance for the LFXT. Nonetheless, an intelligent design of the LFXT target wheel is important for the repetition rate which will be necessary for a future clinical devices. Relevant for the concept of the LFXT is the time it can be operated at maximum dose rate. For this purpose a tungsten target wheel is assumed with radius R that is hit by an electron beam of power *P*.

The focal spot temperature is determined by the surface velocity of the rotating target cylinder and the base temperature of the target surface. The target may be at room temperature initially. The base temperature increases with each revolution of the target. To calculate the base temperature increase a constant heating of the target surface may be assumed. The target temperature increase is simulated by solving the heat equation numerically in Matlab using cylindrical coordinates,

11 \begin{align} \newcommand{\e}{{\rm e}} \displaystyle \frac{\partial T}{\partial t} - \frac{1}{c \rho}\frac{\partial}{\partial r}\left(kr\frac{\partial T}{\partial r} \right) = 0. \nonumber \end{align}

Neumann boundary conditions apply $\partial T/\partial r(r=0) = 0$ and $\partial T/\partial r\left(r = R\right) = P/(Ak)$, where *A* is the area of the target surface hit by the electron beam. Time intervals and space intervals were chosen such that the stability criterion $\Delta t \leqslant c\rho(\Delta x){\hspace{0pt}}^2 / (2k)$ is fulfilled and results become independent of the step size.

### 2.5. Monte Carlo simulations

Monte Carlo simulations were performed in Geant4 version 10.2 using the Penelope low energy physics libraries. The efficiency of electron to photon conversion at a tungsten target was simulated in dependence on the electron beam energy, electron incidence angle and photon emission angle. Similarly the photon spectrum of the source was simulated by scoring generated photons dependent on their kinetic energy. The energy absorption of electrons with depth in the target and their lateral scattering were simulated by binning the absorbed energy in the tungsten material on a fine three dimensional grid with 1 *μ*m side length. The resulting three dimensional dose distribution in the target material was used to determine the focal spot width and the electron penetration depth as defined above.

The achievable focal spot width depends on the possibility to focus the electrons to a focal spot with a high aspect ratio $ \newcommand{\e}{{\rm e}} b/\epsilon$ and on the scattering of the electrons in the target material. In order to calculate the scattering limit of the focal spot size we used Geant4 to simulate an infinitely small beam hitting a tungsten surface perpendicular in a point producing bremsstrahlung as shown in figure 3(b). At an observer point photon trajectories were recorded and the apparent source distribution in the $x^{\prime}-y^{\prime}$ plane calculated.

Microbeam beam profiles were calculated for two conditions

1. Assuming perfectly parallel 50 *μ*m wide and 400 *μ*m spaced microbeams with photon energies according to the spectrum of the biomedical beam line ID17 at the European Synchrotron (ESRF).
2. Microbeams created by the divergent photon field of the MBT with a source size of ${50}~\mu{\rm m} \times {30}~{\rm mm}$ assuming the spectrum of the MBT.

In both cases the beam quality was assessed in a water cube of 0.5 m side length which is placed directly behind the MSC. The microbeam field size was set to $30\times30~{\rm mm^2}$ at the collimator.

### 2.6. Dose rate measurements

The dose rate of a Varian HPX-160-11 industrial x-ray tube was measured using the ionization chambers semiflex TW31010 and farmer TW30002-1 of PTW, Darmstadt, Germany. Dosimetry followed the TRS398 protocoll (IAEA 2000) including detector calibration, temperature and pressure corrections and the application of beam quality factors. Dosimeters were placed in a PMMA phantom of $100\times100\times50~{\rm {mm^3}}$ size at 15 mm depth. The tube was operated at 160 kV and 5 mA, and the beam was filtered by 0.8 mm beryllium and 1 mm aluminum.

## 3. Results

**Figure 5.**
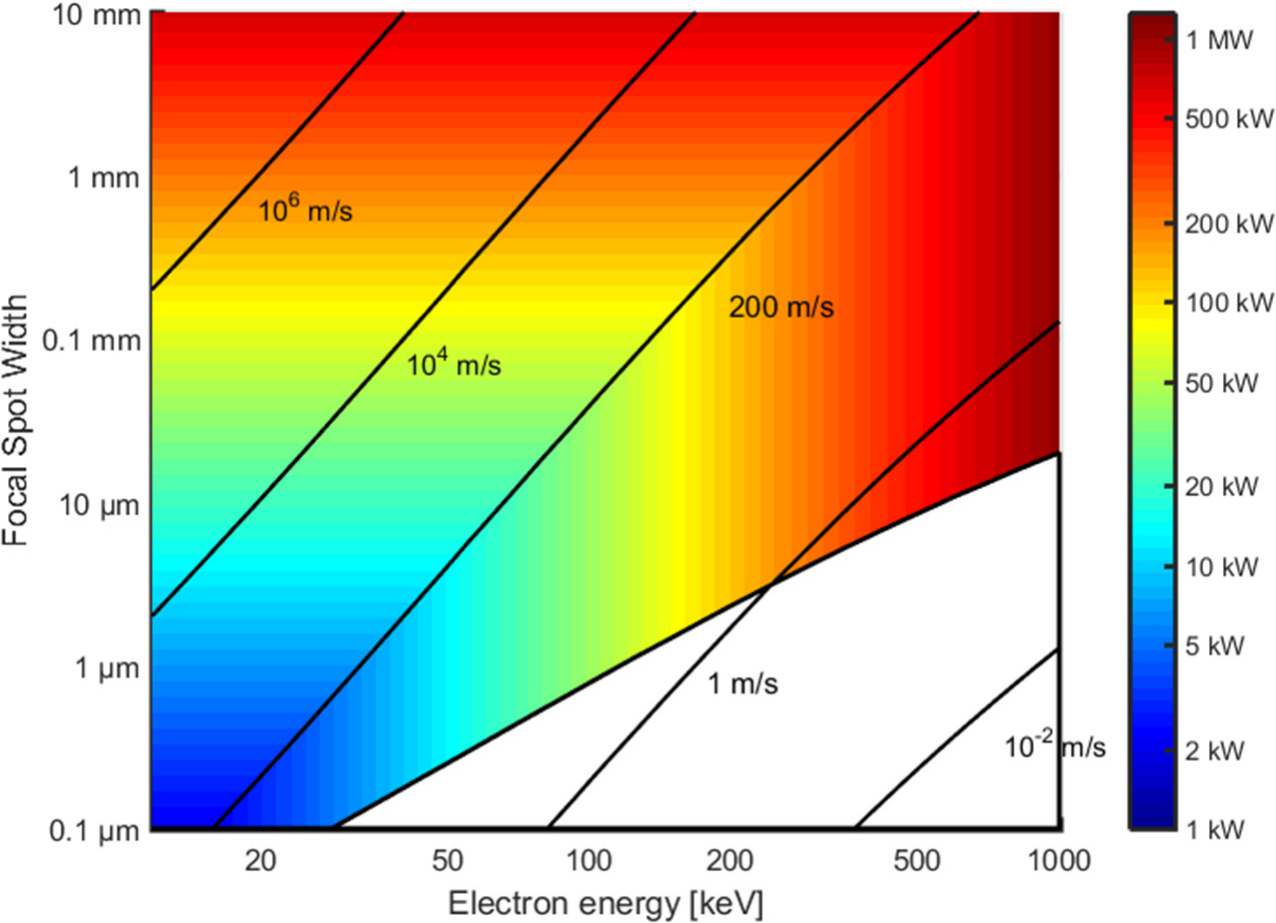
Tube performance at various focal spot widths and acceleration voltages: the maximum possible electron beam power of the x-ray tube is shown in a color scale at a surface velocity of ${200}~{\rm m~s^{-1}}$ and a maximum temperature increase of 2500 K in the focal spot (equations (5) and (2)). The isolines show the transition velocity from heat conduction limit to heat capacity limit (equation (6)).

In figure 5 we show the maximum possible power of an electron beam hitting a rotating tungsten target in a 1 cm long (b) focal spot as a function of the focal spot width ϵ and the electron beam energy which determines the electron penetration depth *d*. The surface velocity of the target is assumed to be $v={200}~{\rm m~s^{-1}}$ and the maximal tolerable temperature increase $\Delta T_{\rm max}={2500}~{\rm K}$. The isolines show the transition velocity *v*_t_. The assumed material parameters density, heat conductivity and heat capacity of tungsten are $\rho={19.3}~{\rm g~cm^{-3}}$, $k={170}~{\rm W~m^{-1}~K^{-1}}$ and $c={138}~{\rm J~kg^{-1}~K^{-1}}$.

While the maximum electron beam power depends on the focal spot width for $v_{\rm t}&gt; {200}~{\rm m~s^{-1}}$ (heat conduction limit), it is independent of the focal spot width for $v_{\rm t}&lt; {200}~{\rm m~s^{-1}}$ (heat capacity limit). However, the maximum electron beam power becomes energy dependent in the heat capacity limit, since it is influenced by the electron penetration depth *d*. A final physical lower limit for the focal spot width is given by lateral electron scattering. This is indicated by the white area at the bottom of the graph with $ \newcommand{\e}{{\rm e}} \epsilon&lt;\epsilon_{\rm min}$.

### 3.1. Electron penetration depth and focal spot widths

**Figure 6.**
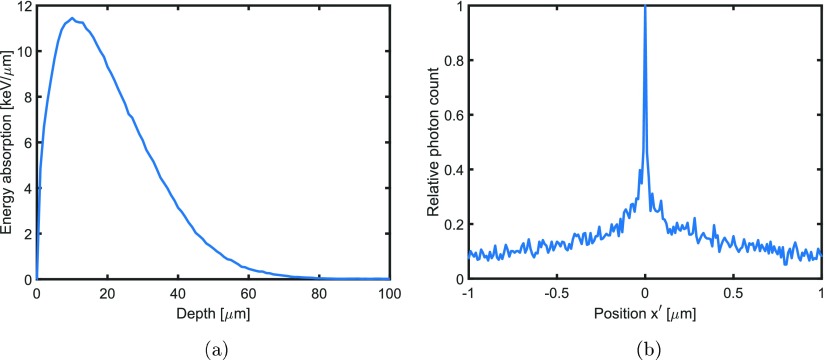
(a) Depicts the energy absorption per depth interval for a 500 keV electron beam in tungsten. The maximum of the energy absorption at around 10 *μ*m depth can be used to calculate the electron penetration depth *d*. (b) Presents the photon emission rate depending on the position $x^{\prime}$ on the target surface (see figure 3(b)).

Figure 6(a) shows the energy absorption per depth interval $\left&lt;\frac{\delta E_{\rm el}}{\delta z}\right&gt;$ of an electron beam with a kinetic energy of 500 keV in tungsten as a function of *z*. The data was obtained from Monte Carlo simulation in Geant4. The maximum of $\left&lt;\frac{\delta E_{\rm el}}{\delta z}\right&gt;$ is used to calculate the electron penetration depth *d*. Table 1 displays *d* for different kinetic energies *E* of the electrons.

**Table 1.** The table shows penetration depths *d* of electrons with different kinetic energies *E* in tungsten.

*E* (keV)	20	50	100	200	500	1000

*d* (*μ*m)	$(0.32\pm0.04)$	$(1.28\pm0.02)$	$(4.00\pm0.3)$	$(12.4\pm0.5)$	$(43.7\pm0.5)$	$(107\pm1)$

Monte Carlo simulations in Geant4 were also used to calculate the minimal achievable focal spot width. The intensity profile of a focal spot hit by an electron beam of electrons with 100 keV kinetic energy in a infinitesimally thin focal spot is shown in figure 6(b). The full width at half maximum (FWHM) of the source is measuring only between 10 and 80 nm. However, due to electron scattering there is a relatively high background noise. Furthermore, there will be technical limitations, such as a finite beam emittance that lead to larger focal spots. More conservatively the source could be defined as the width (FWHM) of the lateral electron scattering which is in the order of $d/3$ (e.g. 1.3 *μ*m in tungsten for 100 keV electrons).

### 3.2. Achievable dose rates and doses in MRT

In order to calculate the performance of the LFXT in MRT we compare the photon flux of the MBT with that of a conventional Varian HPX-160-11 industrial x-ray tube at an acceleration voltage of 160 kV in Monte Carlo simulations. Due to the higher electron to photon conversion efficiency and the harder x-ray spectrum the dose rate of the MBT is $(9.4\pm0.2)$ times higher at equal electron beam power.

For the Varian x-ray tube we measured a dose rate of $(35.3 \pm 1.8)~{\rm m~Gy~s^{-1}}$ at 15 mm depth in water and 30 cm distance from the focal spot at 1 kW electron beam power. Therefore we expect a dose of $(119\pm8)$ m Gy per kJ electron beam energy for the MBT at 50 cm distance from the focal spot. The target surface velocity of the MBT will be ${178}~{\rm m~s^{-1}}$, which allows at the given focal spot size and a maximum temperature increase of 2500 K a maximum beam power of 1.5 MW according to equation (5). Hence the maximum achievable dose rate of the MBT will be around 180 Gy ${\rm s}^{-1}$. An electron beam current of 2.5 A can be obtained from a conventional tungsten filament cathode, provided an appropriate high performance power supply.

**Figure 7.**
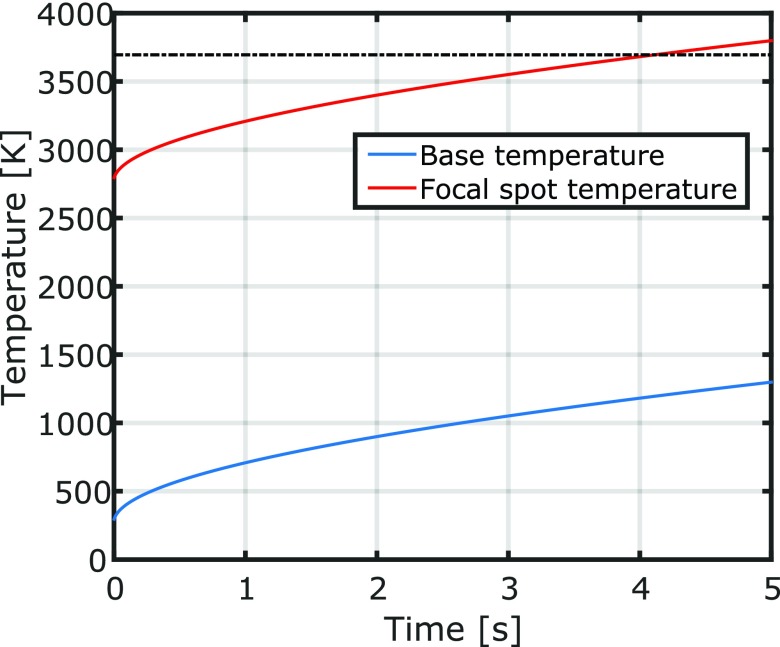
Increase of the target surface temperature with time. The graph shows the base and focal spot temperature of the target cylinder over time as predicted by a finite element calculation of the heat equation. As base temperature we denote the average temperature of the target cylinder surface.

Figure 7 shows the solution of the heat equation, if the heat is equally spread over the target cylinder surface of the MBT. After around 4.1 s the focal spot temperature will exceed the melting point of tungsten. At this time point around 730 Gy have been delivered in 15 mm water depth directly behind the collimator.

**Figure 8.**
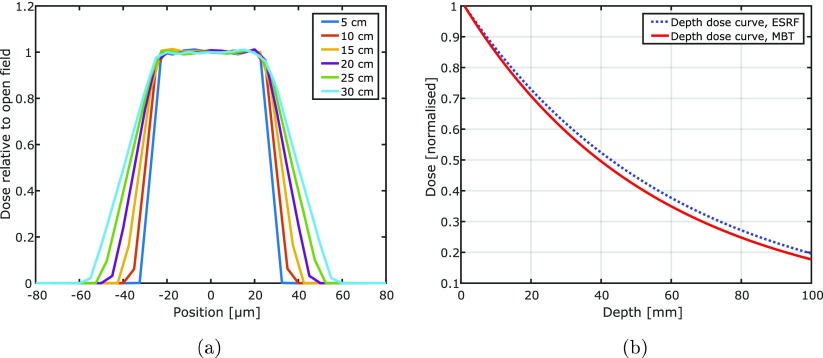
Beam fluence profiles and depth dose curves: (a) shows beam fluence profiles at various distances from the focal spot. The fluence is normalised to the fluence of an uncollimated open field. With distance the beam penumbras increase in width. (b) Compares microbeam depth dose curves at the European Synchrotron (ESRF) and at the MBT.

Apart from the achievable dose rates, beam profiles and depth dose curves of the microbeams produced by the MBT have to be acceptable when aiming to apply the source clinically. Figure 8(a) shows fluence profiles of the MBT at various distances from the collimator surface as calculated in Monte Carlo simulations. The fluence in this graph is normalised to that of an uncollimated open field. Due to beam divergence beam penumbras of the microbeams generated by the MBT are increasing with increasing distance from the collimator. However, even at a distance of 20 cm, the beam penumbras measure only 20 *μ*m in size at a beam to beam spacing of 560 *μ*m. In figure 8(b) we compare the dose fall-off with depth in water of microbeams at the ID17 medical beamline of the European Synchrotron in Grenoble with those produced at the MBT. A shallow dose fall-off is desired, when deep-seated tumours are to be treated.

## 4. Discussion

In this work we present a new concept of brilliant x-ray generation that we term line focus x-ray tubes, LFXT. It is demonstrated that a new generation of highly brilliant x-ray sources based on the LFXT concept can be realized with currently available technical components. Currently, anode surface velocities of ${200}~{\rm m~s^{-1}}$ have already been realized with specialized rotating anode x-ray tubes (Oppelt *et al* 2005) but velocities of ${1000}~{\rm m~s^{-1}}$ seem to be technically feasible. Potential applications of LFXTs could be in phase contrast x-ray imaging and MRT.

**Figure 9.**
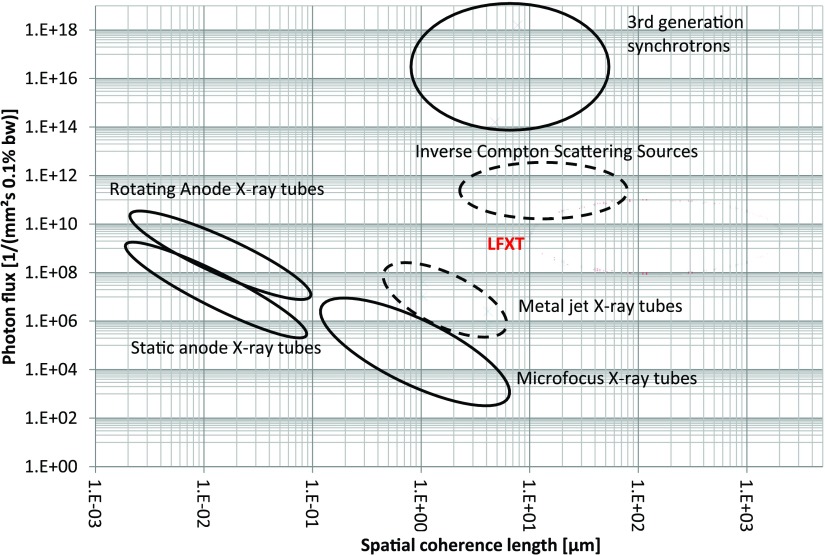
Performance comparison of various x-ray sources: the line focus tube (LFXT) is compared to 3${\rm rd}$ generation synchrotrons, inverse Compton scattering sources and various types of x-ray tubes at a photon energy of 60 keV. Sources shown in a dashed region do usually not reach 60 keV. The realization of the LFXT at very high spatial coherence ($&gt;{100}~\mu$m) depends on the technical feasibility to focus electrons on extremely small focal spots and therefore the area is delineated fainter.

In phase contrast imaging LFXTs may be a promising alternative to current x-ray generation technologies employed for phase contrast imaging ranging from synchrotrons, conventional x-ray tubes with highly absorptive sets of gratings (Pfeiffer *et al* 2006) and inverse Compton scattering sources. In figure 9 we compare the various existing x-ray sources in terms of coherence lengths and photon flux, and show that the LFXT will be a highly competitive technology to facilitate phase contrast imaging. Although spatial coherence is only obtained in one dimension, this would be sufficient for differential hard x-ray interferometers such as Talbot interferometers (Pfeiffer *et al* 2006). The performance estimates are discussed in the appendix.

In contrast to microbeams produced at the European Synchrotron the microbeams produced by the MBT will show beam divergence and a widening of the peak to peak spacing with distance from the multislit collimator. However the beam penumbras remain acceptable small as compared to the beam to beam spacing and the penumbra width measures less than $4\%$ of the beam to beam spacing in 20 cm distance from the collimator. The dose fall-off of the microbeams produced at the European Synchrotron and the MBT is also equivalent. The dose rate at the European Synchrotron is nominally $13\, 000~{\rm Gy~s^{-1}}$, but the field height is only 0.5 mm. In order to generate larger fields the target has to be scanned through the beam. Therefore the effective dose rate for a 3 cm high radiation field is only ${217}~{\rm Gy~s^{-1}}$. Employing a multi-slit collimator (Bartzsch *et al* 2016) at a distance of 0.5 m from the focal spot, we estimate peak entrance dose rates of the MBT in water of up to around ${180}~{\rm Gy~s^{-1}}$ and an acceptable beam divergence. This dose rate is comparable to the effective dose rates at the European Synchrotron (ESRF). Nevertheless the reduced nominal dose rate may affect the microbeam profile, in particular due to cardiovascular motion. Possibly cardiac gating (Chtcheprov *et al* 2014) could be used to avoid motion induced blurring of the dose distributions.

## 5. Conclusions

The LFXT is a new concept of brilliant x-ray production that may help to translate various medical applications of synchrotron radiation such as MRT and phase contrast imaging into clinical practice. Apart from its compact size, the LFXT provides various advantages over synchrotrons such as easier beam control and flexibility in the beam orientation.

Besides the potential to translate the aforementioned technological innovations into clinical practice, LFXTs will potentially revolutionize x-ray applications in various other areas where high brilliance sources are considered to be essential. For instance, in high resolution x-ray imaging, the spatial resolution and the image contrast of small structures are limited by the size of the source and the number of photons absorbed. The LFXT technology may facilitate the fast acquisition of high resolution 2D and 3D images.

When designing a clinical radiation source based on the LFXT technology, various technical details have to be considered. For example the time between repeated exposures in therapy and imaging needs to be kept reasonably small. The various technical challenges will be part of future research. In a next step we plan to develop a prototype based on the presented concepts.

## Figures and Tables

**Figure A1.**
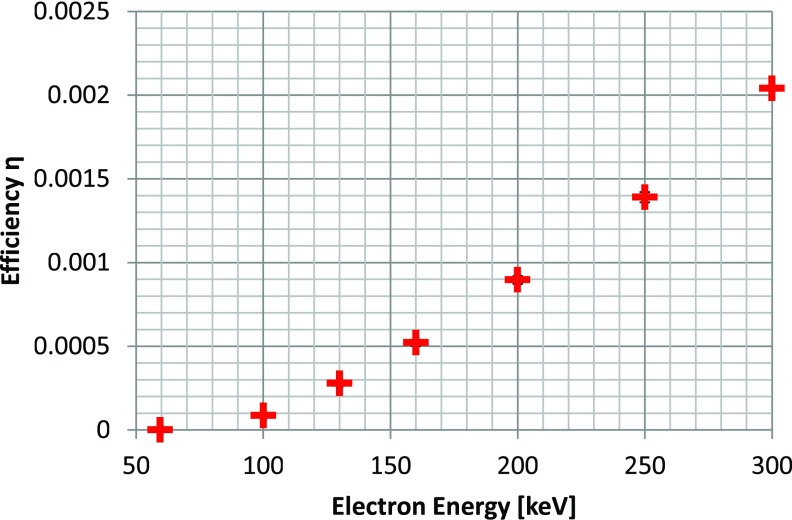
Efficiency of electron to photon conversion in a tungsten target. The conversion efficiency is defined as the number of photons at the tungsten Ka1 fluorescence line emitted per electron. It is zero below and strongly increasing with electron energy above 59.3 keV.

## Appendix. Estimation of photon fluxes and spatial coherences

In figure 9 we compare various sources in terms of coherence length and photon flux. The delineated performance regions are certainly only a rough estimate, though based on data of typical existing sources.

The performance of all x-ray tube types was estimated at the Ka1 absorption edge of tungsten, i.e. at a photon energy of 59.3 keV and a wavelength *λ* of 20.7 pm. The distance r from the source was assumed to be 1 m. For a source with random phase distributions the spatial coherence length *l*_s_ can be approximated by

A.1 \begin{align} \newcommand{\e}{{\rm e}} \displaystyle l_{\rm s} = \lambda\cdot \frac{r}{\epsilon}, \nonumber \end{align}

where ϵ is the source diameter. The flux at distance *r* for an x-ray tube power *P* and acceleration voltage *U* can be calculated from

A.2 \begin{align} \newcommand{\e}{{\rm e}} \displaystyle \Phi = \eta\frac{P}{eU}f_{\Delta\Omega}\cdot\frac{1}{r^2}, \nonumber \end{align}

where *e* denotes the electron charge, *η* the electron conversion efficiency as the number of Ka1 fluorescence photons per electron and $f_{\Delta\Omega}$ is the fraction of photons emitted in a certain angle interval. The electron conversion efficiency is strongly increasing with acceleration voltage *U* for $U &gt; {59.3}~{\rm keV}$ and was calculated in Monte Carlo simulations in Geant4 at various electron energies as shown in figure A1. The fraction of photons emitted per angle interval resembles a completely isotropic source,

A.3 \begin{align} \newcommand{\e}{{\rm e}} \displaystyle f_{\Delta\Omega}\approx 2\cdot10^{-7}~{\rm mrad^{-2}} \nonumber \end{align}

The parameters *η* and $f_{\Delta\Omega}$ will be the same for all x-ray tubes with a tungsten target. Only *P*, *U* and the focal spot size will vary.

As a typical conventional x-ray tube we chose a Varian HPX-160-11 stationary anode x-ray tube with $U = {160}~{\rm kV}$ a focal spot size of 0.4 mm at 800 W or 1.0 mm at 1800 W. This leads to a coherence length of 51.8 nm and 20.7 nm and a photon flux of $3.25\cdot10^{6}~{\rm {mm^{-2}~s^{-1}}}$ and $7.32\cdot 10^6~{\rm {mm^{-2}~s^{-1}}}$ at the small and large focal spot size, respectively.

A typical rotating anode tube is the Siemens Straton Tube with $U = {140}~{\rm kV}$, $P = {100}~{\rm kW}$ and a focal spot size of $1.8\times {7.2}~{\rm {mm^{2}}}$. This leads to a coherence length *l*_s_ of 15 nm in 1 m distance from the source and a photon flux of around $2.8\cdot 10^8~{\rm {mm^{-2}~s^{-1}}}$ (Oppelt *et al* 2005).

Microfocus tubes typically operate at an electron beam power of 4–40 W, (e.g. Hamamatsu microfocus x-ray tube series) at focal spot sizes between 5 and 80 *μ*m with acceleration voltages between 20 and 160 kV. The coherence length is in the order of $0.2$ to 5.0 *μ*m and the photon flux will be between $2\cdot10^4$ and $2\cdot10^5~{\rm {mm^{-2}~s^{-1}}}$.

Metal jet x-ray tubes employ other target materials and therefore the conversion efficiency and the fluorescence lines will be different. As an example we chose Excillum metal jet x-ray tubes. The company claims that the brilliance is between $2.6\cdot10^{10}$ and $10\cdot10^{10}~{\rm s^{-1}~mm^{-2}~mrad^{-2}}$ per spectral line and the source size between 5 and 20 *μ*m. At 5 *μ*m focal spot size the flux will be between $6.5\cdot10^5$ and $2.5\cdot10^6~{\rm {mm^{-2}~s^{-1}}}$. Unfortunately metal jet x-ray tubes operate at lower photon energies. However, to compare coherence lengths we nevertheless assume a wavelength of 20.7 pm which would lead to a coherence length of around 4 *μ*m.

For the inverse Compton Scattering source at the Massachusetts Institute of Technology a beam brilliance of $2\cdot10^{15}\, \frac{1}{\rm {smm^2 ~mrad^2~0.1\%bw}}$ and a source size of 2 times 6 *μ*m are reported (Graves *et al* 2009). At a source distance of again 1 m this infers a photon flux of $2.4\cdot10^{10}~{{\rm {mm^{-2}~s^{-1}}}}$ at a coherence length of 3–10 *μ*m. The best performance is achieved by 3${\rm rd}$ generation synchrotrons with a brilliance between $10^{20}$ and $10^{24} \frac{1}{\rm {smm^2~mrad^2~0.1\%bw}}$ (Huang 2013) and a source diameter of around 50 *μ*m (e.g. Lengeler *et al* (2005)). The distance between source and experiment is usually much larger than 1 m. Therefore we assume, deviating from all previous estimates, a source distance r of 40 m as typically found at the European Synchrotron (ESRF) in Grenoble, France. There the flux is between $10^{14}$ and $10^{18}~{\rm {mm^{-2}~s^{-1}}}$ and the coherence length around 15 *μ*m at a photon energy of 60 keV.
